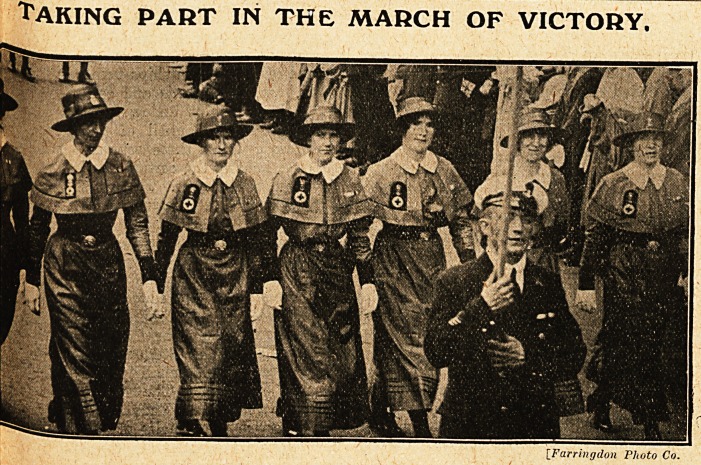# Women in the Victory March, July 19, 1919

**Published:** 1919-07-26

**Authors:** 


### VICTORY DAY IN

### THE MULTITUDE OF UMBRELLAS AT

Photo.]

### WOMEN IN THE VICTORY

Of great and special interest, and a feature of the day to all nurses, was the part taken by the Nursing Services in the great Victory March through London on Saturday, July 19. It was a proud occasion, one to be remembered, and one which must henceforth take a prominent place in the career of each member who had the privilege of taking part in the procession. The heart was thrilled as they came along, by the sight, by the knowledge of them, by the memories they evoked, by the momentous fact of their presence in this pageant of Victors, reminding all of the noble profession of nursing, of its mission to suffering mankind, of its part and place in the history of the war, and in the annals of the nation. "Splendid Women!" ejaculated a man in the crowd as the modest few passed by, too few, if one may venture a criticism, to represent the medical service, and the thousands of other nurses who gave their share in the toils of Active Service at home and abroad. Yet those few were the embodiment of that great and noble work which brought aid and succour to the wounded and stricken soldier; they represented those who had shared with the armies, in every clime, the dire stress and circumstance of war; who through all viscissitudes were ever present with help and cheer, and who now came to share the great triumph of Victory.

Headed by Dame Ethel Becher, G.B.E., R.R.C., Matron-in-Chief, and Dame Maude McCarthy,

### ARMY NURSES WEARING THEIR RIBBONS

Photo ]

### HYDE PARK, 7.50 p.m.
### THE 10,000 VOICES AND MASSED BANDS CONCERT.

[*Topical Press.*

### MARCH, July 19, 1919.

G.B.E., R.R.C., Matron-in-Chief (France), came the "Regulars," or Queen Alexandra's Imperial Military Nursing Service, the premier military service, dignified in bearing, veterans of service, very effective in their scarlet capes and beribboned with service decorations. They were followed by the Civil Hospital Reserve, and the Territorial Force Nursing Service, in scarlet-edged capes and grey dresses. After them came representatives of the Colonial Services, military probationers, and members of the Almeric Paget Massage Corps. At an interval of half-a-dozen paces followed a detachment of V.A.D.s in indoor uniform. A wave of enthusiastic cheering arose as they swung by with a well-measured marching step, and with happy smiling faces. Perhaps the V.A.D.s are better known to the general public, as a section, than the other services, their white caps touching the imagination of the spectators. The Naval Nursing Service were embodied in the Naval contingents, having place between the Anti-aircraft section and the Sea-scouts. They shared the volume of cheering that greeted the Navy along the whole line of the route.

So passed before our eyes a great review, a soul-stirring epoch, a triumph. Following the precept of a great French statesman, let us henceforward turn all our best efforts and energies to the task before us and work. In the nursing of the sick to be re-invigorated, and imbued with the spirit of service; to build up a greater future and strive for the best and give of the best that is in us.

### TAKING PART IN THE MARCH OF VICTORY.

[*Farringdon Photo Co.*

## Figures and Tables

**Figure f1:**
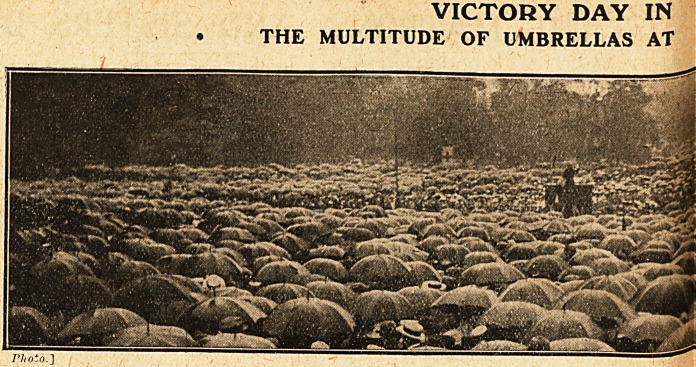


**Figure f2:**
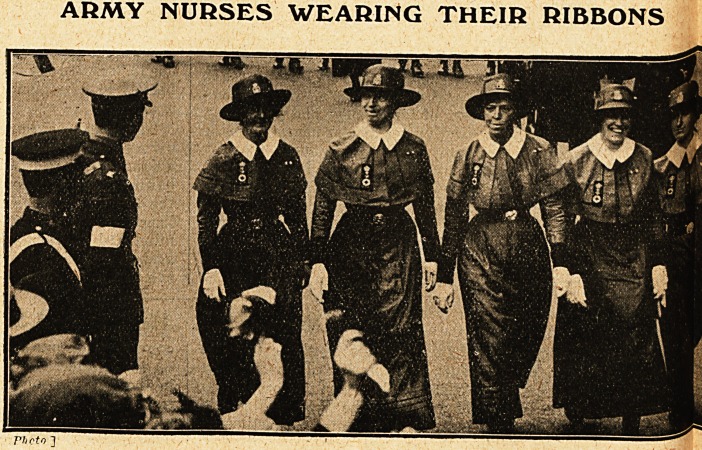


**Figure f3:**
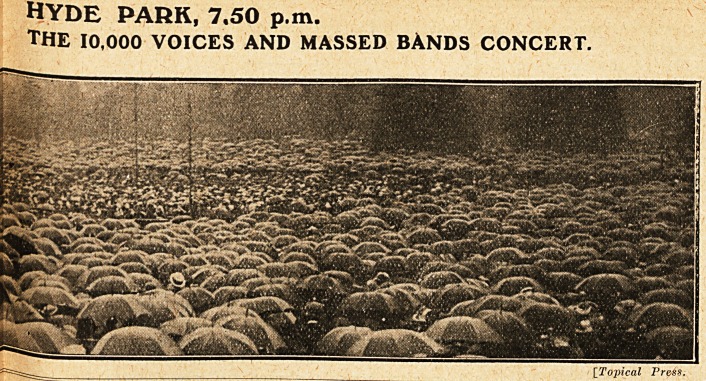


**Figure f4:**